# Patterns of Multimorbidity in Adults: An Association Rules Analysis Using the Korea Health Panel

**DOI:** 10.3390/ijerph17082618

**Published:** 2020-04-11

**Authors:** Yoonju Lee, Heejin Kim, Hyesun Jeong, Yunhwan Noh

**Affiliations:** 1 College of Nursing, Pusan National University, Yangsan 50612, Korea; lyj@pusan.ac.kr; 2 Department of Nursing, The Graduate School, Pusan National University, Yangsan 50612, Korea; pointsun@naver.com; 3 Department of Statistics, The Graduate School, Pusan National University, Busan 46241, Korea; shepd8516@naver.com

**Keywords:** multimorbidity, pattern, association rules analysis, network analysis

## Abstract

This study aimed to identify the prevalence and patterns of multimorbidity among Korean adults. A descriptive study design was used. Of 11,232 adults aged 18 and older extracted from the 2014 Korean Health Panel Survey, 7118 had one or more chronic conditions. The chronic conditions code uses the Korean Standard Classification of Diseases. Association rule analysis and network analysis were conducted to identify patterns of multimorbidity among 4922 participants with multimorbidity. The prevalence of multimorbidity in the overall population was 34.8%, with a higher prevalence among women (40.8%) than men (28.6%). Hypertension had the highest prevalence in both men and women. In men, diabetes mellitus and hypertension yielded the highest probability of comorbidity (10.04%). In women, polyarthrosis and hypertension yielded the highest probability of comorbidity (12.51%). The results of the network analysis in four groups divided according to gender and age showed different characteristics for each group. Public health practitioners should adopt an integrated approach to manage multimorbidity rather than an individual disease-specific approach, along with different strategies according to age and gender groups.

## 1. Introduction

Multimorbidity is defined as the co-occurrence of two or more diseases in the same person [1]. The number of chronically ill patients and the prevalence of multimorbidity are increasing due to the rising aging population worldwide [2,3]. Researchers have investigated the prevalence of multimorbidity across countries or regions such as the United States, Australia, Canada, Europe, the Netherlands, Iran, and Mexico, and found that its prevalence varies widely across studies from 12.9% to 95.1% [4,5,6,7,8,9,10,11]. A meta-analysis of research based on 70 community-based studies demonstrated that the overall pooled prevalence of multimorbidity was 33.1% [12]. The burden of medical expenses is growing with the increase in the prevalence of multimorbidity due to the rise in the aging population [13].

The wide variation on the prevalence, pattern and risk factors of multimorbidity is due to age and sex [9,14], source of information [15,16], analysis method [17]. In addition, varying definitions of multimorbidity [15] and the classification system of non-communicable disease [18] makes it difficult to compare study results. Medical expenses due to multimorbidity also influence the medical delivery system and the payment system for medical expenses [13].

Multimorbidity is often associated with functional limitation, reduced quality of life, higher mortality, polypharmacy and high treatment burden, higher rates of adverse drug events, and frequent use of health services [19,20]. The National Institute for Health and Care Excellence (NICE) [21] recommended that the multimorbidity approach should go beyond the benefits and risks of the guidelines for single health conditions, while focusing on the interaction between health conditions and treatment and their effect on quality of life.

The Korean healthcare system is dominated by the private sector instead of the primary care sector. Patients can visit any specialty clinic without referrals from family physicians (self-referral) or with referrals from other specialists (cross-referral) [22,23]. The highly fragmented model of specialist services is limited to effectively deal with the challenges of an aging population and non-communicable diseases [24]. Effective and efficient health care aimed at improving the health status and quality of life of people affected by multimorbidity requires a new integrated and innovative treatment model instead of the existing individual chronic disease-specific approach [7]. Understanding the prevalence of multimorbidity, the combination patterns of diseases, and the association between non-communicable disease in Korea should, therefore, be given priority.

Only a few studies on multimorbidity have been conducted in Korea. A study based on the Health Insurance Review and Assessment Service’s patient sample [24] identified that the prevalence of multimorbidity was 24.6% for outpatients over the age of 19. The prevalence of multimorbidity was higher in women than men and it increased with age. The most common multimorbidity diseases were hypertension, gastritis, muscular diseases, allergic diseases in all subjects, diabetes in men, and musculoskeletal diseases in women. The results also showed that the most common multimorbidity diseases in the population under the age of 45 were gastritis, allergic diseases, and musculoskeletal diseases; however, hypertension, and diabetes were the most prevalence multimorbidity diseases in subjects over the age of 45. Another study based on the Korean Health Panel Survey [13] has shown similar results. The prevalence of multimorbidity in the adult population over the age of 20 was 39.6%, whereas the prevalence of multimorbidity in the population over the age of 65 was 66.7%. The most common dyad and triad of chronic conditions for the nonelderly patients were hypertension-diabetes, hypertension-arthritis-osteoporosis and, for the elderly, they were hypertension-arthritis, hypertension-hyperlipidemia-arthritis. Although multimorbidity is strongly associated with socio-demographic factors, there is a limited understanding of the association between diseases, and gender and age patterns of multimorbidity. Moreover, these studies suggested the prevalence of multimorbidity based on 20 specific chronic diseases presented by the US Office of the Assistant Secretary of Health and the prevalence of two or three high-frequency disease associations. Therefore, there is limited understanding of the association between diseases, and gender and age patterns of multimorbidity.

In terms of analytical methods, the epidemiological studies tend to use cluster analysis [7], principal component analysis [17], and simple prevalence [25] to simplify and interpret data [26,27]. Association Rule Mining (ARM), a data mining technique used extensively in healthcare [26,28], attempts to discover and predict rules by extracting simple structures from a set of items in a database [29]. Network-based approaches to human disease can have multiple biological and clinical applications, and network graphics help to identify highly connected chronic conditions in multimorbidity networks [30,31].

In summary, although researchers have examined the prevalence of multimorbidity and its association with frequency disease has been examined, few studies have focused on multimorbidity networks by age and gender. The purpose of this current study was to identify the prevalence of multimorbidity and compare the patterns by age and gender in Korea using association rule analysis.

## 2. Materials and Methods

### 2.1. Data Source

The Korea Institute for Health and Social Affairs (KIHASA) and the National Health Insurance Service (NHIS) formed a consortium in 2008 and have been jointly conducting KHPS to generate basic data on health status, health services usage, healthcare expenditures, and health behaviors.

The KHPS uses 90% of the 2005 Population and Housing Census data as its sampling frame in order to maintain national representativeness. Sample households were chosen through the following processes: (1) select sample districts (cluster); (2) choose sample households in enumeration districts, using a probability proportionate and stratified cluster sampling method. With the total number of enumeration districts being 350, the initial study participants included 7866 households and their 24,616 family members.

The KHPS collects data and information using computer assisted personal interviewing (CAPI) technique on the following: demographic characteristics, income, savings and expenses, employment, housing, chronic conditions, use of medical services, medication, charges and source of payments, private health insurance, pregnancy and delivery, elderly care, health behaviors and health awareness.

### 2.2. Measures

The KHPS included self-report measures written in Korean to assess past and current chronic conditions, regardless of medical diagnosis, and trained interviewers converted the chronic conditions into the chronic disease codes which were based on the Korean Standard Classification of Diseases (KCD-6). KCD-6 is modified national versions of the International Statistical Classification of Diseases and Related Health Problems (ICD-10). It was adopted for diagnosis and procedure coding to facilitate the submission of medical billing and reimbursement by health insurers. KCD is divided into 22 chapters, 264 blocks, and 2049 three-character codes. In this study, the blocks (disease categories) were used to identify prevalence and patterns of multimorbidity in the adult population. Demographic characteristics including age, marital status, education level, disability, occupational status, and number of chronic conditions were also analyzed.

### 2.3. Study Process and Subjects

This study was approved by the Institutional Review Board (IRB; No. 2017_124_HR) with a waiver for informed consent because the data were obtained from a public database.

The raw data were provided by the Korea Health Panel website (www.khp.re.kr) through an application procedure. The KHPS includes an identification number for each household and each member; however, the number is not associated with any personal information. The data were derived from the 2008–2014 KHPS (version 1.1). The data for 2014 included 5001 households consisting of 13,973 family members. Of these, 11,232 participants were 18 or older, 7118 participants had at least one chronic condition, and 4922 participants had two or more chronic conditions. Participants with multimorbidity were included in the association rule and network analysis.2.4. Statistical Analysis.

The prevalence of chronic conditions was analyzed with descriptive statistics using frequency and percentages. Gender differences in the demographic characteristics of participants were compared using a Rao-Scott Chi-square test for complex samples. Sample weights for the KHPS were calculated after going through the process of adjusting for unequal selection probabilities and non-responses and making a population distribution disclosure via post-stratification corresponding to the sample distribution. The cross-sectional weight for household members was used.

Since more than 200 chronic conditions were included, the scope for identifying significant associations was limited. Therefore, consistent with previous research [17,32], only diseases with a prevalence greater than 1% in each gender were considered to obtain clinically interpretable and significant association patterns.

To identify the pattern of multiple chronic conditions, an association rule analysis was performed on pairs of diseases with high prevalence. Association rule analysis finds associations between two or more items in an event [29]. Association rules commonly use support, confidence, and lift as measurement ratios. Support refers to the probability that a particular disease A and B occur at the same time, while confidence refers to the ratio of occurrence of disease A and B at the same time A occurs [33], and lift is the ratio of the observed support to that expected if A and B were independent. A lift of ‘1′ means that the probability of occurrence of the antecedent and that of the consequent are independent of each other. Hence, a higher lift indicates a higher chance of co-occurrence of the consequent with the antecedent and a more significant association [33]. This study determined the minimum threshold for each variable, with measures of support and confidence greater than 10% and lift greater than one.

Furthermore, social network analysis was conducted to identify the association between fifteen frequent diseases among men and women. Centrality analysis was used to identify the relationship between the core chronic conditions of the network structure and diseases that appear simultaneously. We measured degree, betweenness, and closeness as indexes of centrality [34]. In the network, the degree of centrality of disease represents a direct association with other diseases. The betweenness of disease determines its mediating role in the network. The closeness of a disease indicates the number of steps from other diseases in the network. A higher closeness indicates a higher risk of being diagnosed with the associated disease with fewer steps. Since we do not aim to identify causality of multimorbidity, a network between two diseases is created when appearing at the same time with no direction. Further, the diameter of the node is proportional to the prevalence of the chronic disease, and the thickness of the edge demonstrates the strength between two linked diseases.

All the statistical tests were performed using SPSS 23.0 (IBM, Armonk, NY, USA), with a 0.05 level of significance. Association rule and network analysis and its visualization were done using the R 3.4.0 (The R Foundations for Statistics and Mathematics, Vienna, Austria) with the arules package and the arulesViz package.

## 3. Results

### 3.1. Characteristics of the Participants

Out of 11,232 adults, 49.6% were men. The average age was 56.78 ±16.04 years and 58.21 ± 16.11 years for men and women, respectively, and over 80% of the participants were under 65. The characteristics of the participants by gender are shown in Table 1. All characteristics except marital status showed statistically significant differences between men and women.

The differences in characteristics of participants with or without multimorbidity are shown in Table 2. The prevalence of multimorbidity in the overall population was 34.8%, with a higher prevalence in women (40.8%) than in men (28.6%). The prevalence of multimorbidity in participants aged 65 or older was 85.2%, which is more than three times the prevalence among participants under 65. There were statistically significant differences in all variables including marital status, education level, disability, and occupational status among participants with or without multimorbidity.

### 3.2. Prevalence of Multimorbidity

**Figure 1:**
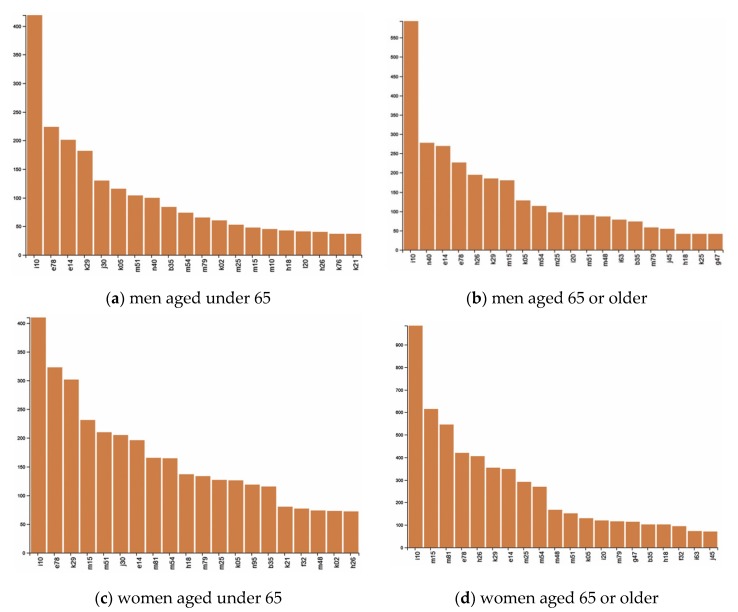
Prevalence of chronic conditions by gender and age. (**a**) men aged under 65; (**b**) men aged 65 or older; (**c**) women aged under 65; and (**d**) women aged 65 or older; Note: B35 = Dermatophytosis, E14 = Unspecified diabetes mellitus, E78 = Disorders of lipoprotein metabolism and other lipidemias, F32 = Depressive episode, G47 = Sleep disorders, H18 = Other disorders of cornea, H26 = Other cataract, I10 = Essential (primary) hypertension, I20 = angina pectoris, I63 = Cerebral infarction, J30 = Vasomotor and allergic rhinitis, J45 = Asthma, K02 = Dental caries, K05 = Gingivitis and periodontal diseases, K21 = Gastroesophageal reflux disease, K25 = Gastric ulcer, K29 = Gastritis and duodenitis, L20 = Atopic dermatitis, M10 = Gout, M15 = Polyarthrosis, M25 = Other joint disorders, M48 = Other spondylopathies, M51 = Other intervertebral disc disorders, M54 = Dorsalgia, M75 = Shoulder lesions, M79 = Other soft tissue disorders, M81 = Osteoporosis without pathological fracture, N40 = Hypertrophy of prostate, N95 = Menopausal and other perimenopausal disorders.

Figure 1 shows the frequency of diseases according to gender and age, with age classified as under 65 and 65 or older. Primary hypertension (HTN) and disorders of lipoprotein metabolism and other lipidemias (dyslipidemias) were the top ranked diseases among all age groups. Top ranked diseases among participants aged under 65 included rhinitis, other intervertebral disc disorders (intervertebral disc disorder), dermatophytosis, while cataract, polyarthrosis, and other point disorders were top ranked among participants aged 65 or older. Prostatic Hyperplasia (PH) was ranked eighth among men under 65, and second among men aged 65 or older. Gastritis and duodenitis were ranked third among women under 65, and osteoporosis without pathological fracture was ranked third among women aged 65 or older. Intervertebral disc disorders, rhinitis, and disorders of the cornea were included in the top 10 in the under 65 group, while cataract, joint disorders, and spondylopathies were included in 65 or older group. HTN and dyslipidemias were ranked first and second in both men and women under 65.

### 3.3. Patterns of Multimorbidity

#### 3.3.1. Association Rules and Frequent Set Analyses

Table 3 shows the results of the association rule analysis by gender. Among men, the probability for DM and HTN was 10.04%, dyslipidemias and HTN was 9.97%, and PH and HTN was 6.97%. Further, 65.33% of people with dyslipidemias also reported HTN, whereas 26.14% of those with HTN also reported dyslipidemias. Thus, people with dyslipidemias are more likely to have two chronic conditions at the same time than people with HTN. Similarly, 61.22% of those with DM also reported HTN, and 26.31% of those with HTN also had DM, indicating a difference in confidence between the two diseases.

Among women, the probability for polyarthrosis and HTN was 12.51%, dyslipidemias and HTN was 12.36%, and osteoporosis without pathological fracture and HTN was 9.88%. Among participants with DM, 70.27% also reported HTN. Conversely, 26.40% of participants with HTN also reported DM, which indicated that participants with DM are more likely to have two chronic conditions at the same time than those with HTN.

#### 3.3.2. Network Analyses

**Figure 2:**
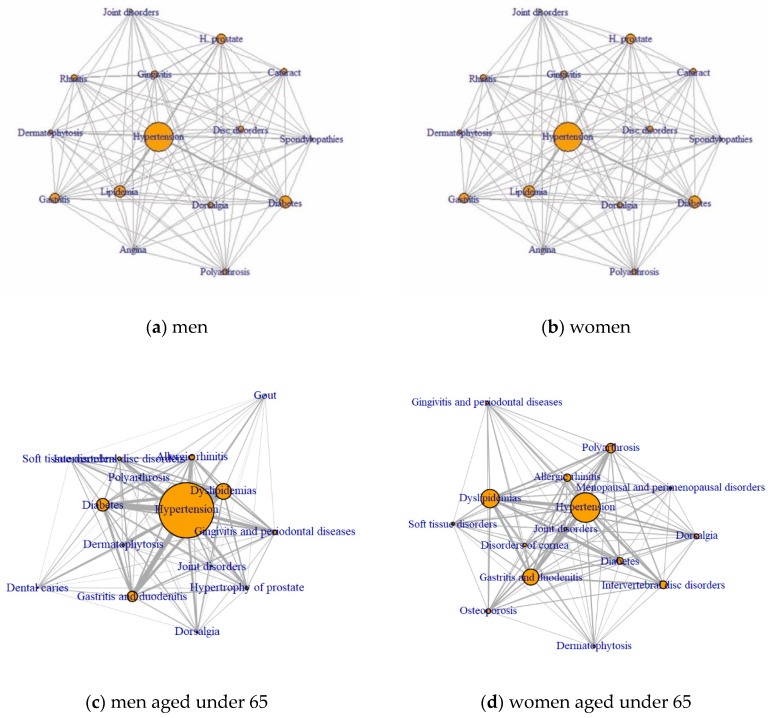

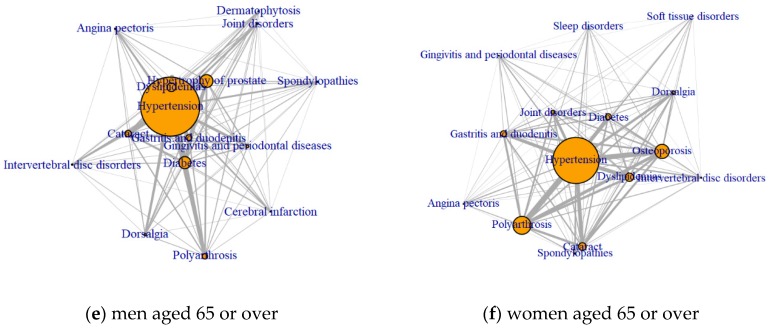
Network of frequent disease based on centrality by gender and age. (**a**) men; (**b**) women; (**c**) men aged under 65; (**d**) women aged under 65 (**e**) men aged 65 or older; and (**f**) women aged 65 or older.

The results of the visualization of each node’s influence within the network using centrality analysis are shown in Figure 2. For men, the diseases with degree centrality of more than 0.5 included HTN, PH, and dyslipidemias. HTN reported the highest betweenness centrality followed by DM, and other intervertebral disc disorders. The closeness centrality of the top 15 diseases was less than 0.00014 and with similar values.

For women, the diseases with a degree centrality more than 0.5 included HTN, osteoporosis without pathological fracture, dyslipidemias, gastritis, polyarthrosis, and DM. Betweenness centrality measures appeared in the following order: osteoporosis, dyslipidemias, polyarthrosis, and joint disorder. Similar to men, closeness centrality was very small, 0.00017 or less, and the top 15 diseases reported similar values.

For men aged under 65, HTN, dyslipidemias, DM, gastritis, and allergic diseases were the top five diseases of degree centrality. For women aged under 65, HTN, dyslipidemias, gastritis, polyarthritis, and other intervertebral disc disorders were the top five diseases of degree centrality. For men aged 65 or over, HTN, PH, DM, dyslipidemia, and cataract were the top five diseases of degree centrality. For women aged 65 or over, HTN, polyarthritis, osteoporosis, dyslipidemias, and cataract were the top five disease of degrees centrality.

## 4. Discussion

This study was conducted to comprehensively identify the prevalence of multimorbidity and evaluate the relationships between morbidities based on the association rules analysis in the Korean adult population by gender and age. For men aged under 65, HTN, dyslipidemias, DM, gastritis, and allergic diseases were the top five diseases of degree centrality. For women aged under 65, HTN, dyslipidemias, gastritis, polyarthritis, and other intervertebral disc disorders were the top five diseases of degree centrality. For men aged 65 or over, HTN, PH, DM, dyslipidemia, and cataract were the top five diseases of degree centrality. For women aged 65 or over, HTN, polyarthritis, osteoporosis, dyslipidemias, and cataract were the top five diseases of degree centrality.

### 4.1. Relationship with Existing Literature

The findings of the current study were compared with previous studies based on similar definition of multimorbidity in the general adult population because the prevalence may differ depending on population characteristics, including data source and size, age range, ethnicity, and definition of multimorbidity. The prevalence rates estimated in the United States [9], Australia [16], and Iran [14] were lower than the prevalence of the present study. Rocca et al. [9] suggested lower prevalence of multimorbidity among individuals of Asian origin; our results are inconsistent with this [9]. Mexican research studied patients whose ages ranged from 25 to 75 years at a primary care clinic reported that the age-standardized prevalence of multimorbidity was 69.5% [7], while a study based on adults at a primary care center in Portugal estimated a prevalence rate of 72.7% [30], which were both higher than the prevalence of multimorbidity estimated in the present study. Since these studies analyzed data collected from primary care settings, these variations might be due to the setting and method of data collection [27].

The present findings suggest a higher prevalence among women compared to men, and a positive relationship between prevalence and age. Additionally, a study using patient sample data provided by the Korean Health Insurance Review & Assessment Service, reported a prevalence of 81.2% in patients over 65 [35]. Furthermore, estimating the prevalence of multimorbidity in a country is necessary while developing effective health care policies for management of multimorbidity.

A significant relationship (*p* < 0.001) between demographic characteristics and the presence of multimorbidity was identified. Therefore, socio-demographic factors of patients with multimorbidity should be assessed when developing public nursing programs in primary care settings.

HTN was the most prevalent disease among men and women, regardless of age [8,31,36,37]. The prevalence of HTN and dyslipidemias were the highest in men and women under 65. The analysis revealed gender differences in the frequency of diseases. The highest absolute frequencies are determined by the prevalence rates of each disease in combinations. For example, given its high prevalence in the population, hypertension is a part of the most frequent disease combinations [38].

Therefore, it is necessary to view the pattern of hypertension from the perspective of the nonrandom association of health problems. The prevalence of diseases of the circulatory system (Code I of KCD-6) was higher among men (15.4%) compared to women (12%). Conversely, the prevalence of diseases of the musculo-skeletal system and connective tissue (Code M of KCD-6) was considerably high among women (25.5%) compared to men (15.2%). In addition, excluding genital disorders, cerebral infarction, asthma, gout, and gastric ulcer yielded a prevalence of 1% or more in men, which may relate to poor health habits such as smoking, alcohol consumption, and obesity. Conversely, depressive episodes, sleep disorders, gastroesophageal reflux disease, and osteoporosis yielded a prevalence of 1% or more among women, which may be due to changes in characteristics, diet and hormones of emotionally sensitive women [39].

The results of the association rule analysis provide probabilities of pair-wise combinations of morbidities that occur more frequently than expected by random chance [26]. These results may help healthcare providers in recognizing the risk of subsequent diseases while assessing patients without multimorbidity, and in determining intervention and prevention strategies to prevent occurrence of multimorbidity [37]. For example, the results revealed that the pair of diseases with the highest lift, among both men and women, was DM dyslipidemias. Similar measures of support and lift measures greater than two indicate that participants diagnosed with one of the two diseases had a 30% chance of developing the other, which is not coincidental but correlated. In addition, people with dyslipidemias are more likely to have two chronic diseases at the same time than people with HTN; thus, patients with dyslipidemias should engage in activities that prevent HTN. Similarly, in women, the probability of HTN when DM, cataract and point disorder were present, was higher than the probability of other diseases when HTN was present. These results suggest that HTN should be assessed in the presence of these diseases.

The results of the network analysis revealed that HTN had the highest degree centrality among men and women, which suggests that HTN was the most influential in the network consisting 15 most frequent diseases. HTN may be most frequently associated with other diseases in the network due to its high prevalence rate in this study. PH, especially among men, is frequently linked to HTN, DM and dyslipidemias.

HTN yielded the highest measures of betweenness centrality in both genders, which indicates that HTN may act as a mediator between unrelated diseases. There is a particular point to be noted while interpreting degree centrality and betweenness centrality. For example, DM presented the second highest betweenness centrality among men, while dyslipidemia presented the second largest degree centrality, but was lower than DM in betweenness centrality. Thus, DM is less likely to be associated with other diseases directly than dyslipidemia, but DM plays a greater role in mediating the simultaneous occurrence of other diseases. HTN yielded the highest mediated centrality among women, which was 1.4 times higher than for men. In addition, there were eight other diseases among women which presented a mediated centrality greater than 2000 as compared to only HTN in men. In particular, women reported a high prevalence of musculoskeletal diseases, with a high probability of co-occurrence with other diseases in the rules of association and a high degree of centrality in the network. Hagen et al. [40] suggested that individuals with HTN reported a lower prevalence of chronic musculoskeletal conditions compared to individuals with normal BP, which indicates that musculoskeletal disorders may be difficult to detect early among people with HTN. This study highlights the need to assess the presence of musculoskeletal symptoms among patients at primary care clinics or public health centers to manage HTN.

A high closeness centrality of a particular disease in the network acts as a hub. However, our results revealed that the measures of closeness centrality for all diseases in men and women were very small and relatively constant. Thus, no particular disease acted as a hub, and most diseases depicted a similar pattern to that appears with other diseases.

The results of the network analysis in four groups divided by gender and age showed different characteristics for each group. Hypertension and dyslipidemia had a high degree of centrality in all groups. Gastritis and allergic disease had a high degree of centrality in men aged under 65, and gastritis, polyarthritis, and disc disorder had a high degree of centrality in women aged under 65. PH, DM, and cataract had a high degree of centrality in men aged over 65, and polyarthritis, osteoporosis, and cataract had a high degree of centrality in women aged over 65. These results were similar to those of previous studies, in which the most common multimorbidity was DM in men, musculoskeletal diseases in women, and gastritis, allergic diseases, and musculoskeletal diseases in the population aged under 45, hypertension and diabetes in those over the age of 45 [13]. In this study, instead of analyzing gender and age separately, considering the gender and age together, it was confirmed that the characteristics of the multimorbidity to be considered in each group are different.

The present findings suggest gender and age differences in the prevalence of specific disease, number of morbidities experienced, and pattern of combinations, with a higher prevalence of diseases and greater centrality. This suggests that treatment of multimorbidity requires an integrated approach instead of an individual disease-specific approach. Therefore, sharing medical information among fragmented disease specialists is very important for the successful treatment of complex diseases [36]. In Korea, public health nurses provide non-communicable diseases management for the community population, with a focus on managing HTN, DM, and metabolic diseases. In this study, HTN, DM and metabolic diseases were frequently paired together, and HTN was highly associated with other diseases. Therefore, it is necessary to identify the obstacles to health promotion by assessing the concurrent disease and the medication history while designing interventions and non-communicable diseases management projects.

Effective treatment of multimorbidity may require joint consultation with specialists, who treat individual diseases, in order to identify and treat other coexisting conditions. An information technology system that can track and share patient health records between various individual health systems may be effective in managing multimorbidity [36]. A primary, care-centered service model for treatment of chronic diseases that creates a link between clinics and community health centers, by expanding services to integrate education and treatment, and maintaining patient records, may also be effective [37].

### 4.2. Limitations and Implications for Future Study

Since the type and number of diseases as criterions for multimorbidity diagnosis have not been limited, the findings have the strength of providing a broad perspective on the combination of diseases, as compared to previous studies. Nevertheless, there are some limitations to this study. The present study excluded people living in nursing homes. Since past findings [15] state that the rate and pattern of the multimorbidity may differ depending on population, it is necessary to expand the scope of participants to conduct comparative studies. Data collection using self-report measures may not be accurate in estimating trends in multimorbidity; thus, future research should be based on medical diagnosis or medical record analysis to minimize variation. Since this study was cross-sectional the findings could not prove causal relationships between diseases. Moreover, longitudinal studies should be conducted to identify risk factors and the pattern of specific complex diseases to prevent the occurrence of multimorbidity. The research cited in this paper only investigated the prevalence and association of multimorbidity. Future studies should investigate risk factors for specific combinations of diseases and develop management guidelines for these specific multimorbidity patterns.

## 5. Conclusions

This is a cross-sectional study to identify the prevalence and the patterns of multimorbidity in the Korean general populations aged 18 or older. The results of the network analysis in four groups divided by gender and age showed different characteristics for each group. Hypertension and dyslipidemia had a high degree of centrality in all groups. Gastritis and allergic disease had a high degree of centrality in men aged under 65, whereas gastritis, polyarthritis, and disc disorder had a high degree of centrality in women aged under 65. PH, DM, and cataract had a high degree of centrality in men aged over 65, whereas polyarthritis, osteoporosis, and cataract had a high degree centrality in women aged over 65. Because of the patterns of the multimorbidity according to gender and age, an integrated approach to manage multimorbidity rather than an individual disease-specific approach, along with different strategies according to age and gender group, should be separately developed to prevent multimorbidity. The research works cited in this paper only investigated the prevalence and association of multimorbidity. Future studies should investigate risk factors for specific combinations of diseases and develop management guidelines for these specific multimorbidity patterns.

## Figures and Tables

**Table 1:** Participant characteristics and the differences among sex. (N ^1^ = 11,232).

Variables	Categories	Sex, n ^1^ (%) ^2^				*F*	(*p*)
		Men (n ^1^ = 5339)		Women (n ^1^ = 5893)			
Age	<65	4133	(87.5)	4264	(82.6)	68.83	(<0.001)
	≥65	1206	(12.5)	1629	(17.4)		
Marital status	Spouse	3737	(64.2)	3742	(63.2)	0.99	(0.320)
	Spouseless	1602	(35.8)	2151	(36.8)		
Education	Elementary school or lower	665	(8.0)	1672	(19.6)	122.55	(<0.001)
	Middle school	528	(7.6)	688	(10.0)		
	High school	1707	(31.2)	1652	(30.3)		
	College degree or higher	2439	(53.2)	1881	(40.1)		
Disability	Yes	464	(6.9)	363	(4.7)	24.02	(<0.001)
	No	4875	(93.1)	5530	(95.3)		
Occupational status	Yes	3700	(72.2)	2847	(50.7)	445.64	(<0.001)
	No	1639	(27.8)	3046	(49.3)		
Number of chronic diseases	0	2241	(49.9)	1873	(38.9)	49.61	(<0.001)
	1	1121	(21.5)	1075	(20.3)		
	2	664	(11.3)	745	(12.3)		
	3	471	(7.1)	603	(8.8)		
	4	331	(4.4)	527	(7.1)		
	≥5	511	(5.8)	1070	(12.6)		

^1^ Unweighted N; ^2^ Weighted %.

**Table 2:** Differences in characteristics of participants with or without multimorbidity. (N ^1^ = 11,232).

Variables	Categories	Chronic Diseases, n ^1^ (%) ^2^				*F*	(*p*)
		Non-Multimorbidities		Multimorbidities			
		(n ^1^ = 6310)		(n ^1^ = 4922)			
Sex	Men	3362	(71.4)	1977	(28.6)	163.25	(<0.001)
	Women	2948	(59.2)	2945	(40.8)		
Age	<65	5917	(74.1)	2480	(25.9)	2852.76	(<0.001)
	≥65	393	(14.8)	2442	(85.2)		
Marital status	Spouse	3895	(59.8)	3584	(40.2)	234.50	(0.001)
	Spouseless	2415	(74.9)	1338	(25.1)		
Education level	Elementary school or lower	426	(21.7)	1911	(78.3)	745.96	(<0.001)
	Middle school	378	(37.1)	838	(62.9)		
	High school	2015	(64.6)	1344	(35.4)		
	College degree or higher	3491	(83.9)	829	(16.1)		
Disability	Yes	194	(30.4)	633	(69.6)	322.85	(<0.001)
	No	6116	(67.4)	4289	(32.6)		
Occupational status	Yes	4112	(70.1)	2435	(29.9)	161.32	(<0.001)
	No	2198	(57.6)	2487	(42.4)		

^1^ Unweighted N; ^2^ Weighted %.

**Table 3:** Association rules analysis of chronic conditions.

Rule				Support (%)	Confidence (%)	Lift
Men						
1	Diabetes	==>	Hypertension	10.04	61.22	1.60
2	Hypertension	==>	Diabetes	10.04	26.31	1.60
3	Dyslipidemias	==>	Hypertension	9.97	65.33	1.71
4	Hypertension	==>	Dyslipidemias	9.97	26.14	1.71
5	Prostatic hyperplasia	==>	Hypertension	6.97	54.68	1.43
6	Hypertension	==>	Prostatic hyperplasia	6.97	18.27	1.43
7	Gastritis	==>	Hypertension	5.10	38.35	1.01
8	Hypertension	==>	Gastritis	5.10	13.37	1.01
9	Diabetes	==>	Dyslipidemias	5.10	31.10	2.04
10	Dyslipidemias	==>	Diabetes	5.10	33.40	2.04
11	Cataract	==>	Hypertension	4.49	55.82	1.46
12	Hypertension	==>	Cataract	4.49	11.76	1.46
13	Gingivitis	==>	Hypertension	3.97	40.73	1.07
14	Hypertension	==>	Gingivitis	3.97	10.41	1.07
15	Polyarthrosis	==>	Hypertension	3.78	45.17	1.18
16	Hypertension	==>	Polyarthrosis	3.78	9.90	1.18
17	Disc disorders	==>	Hypertension	3.39	39.92	1.05
18	Hypertension	==>	Disc disorders	3.39	8.88	1.05
19	Prostatic hyperplasia	==>	Dyslipidemias	3.10	24.30	1.59
20	Dyslipidemias	==>	Prostatic hyperplasia	3.10	20.30	1.59
Women						
1	Polyarthrosis	==>	Hypertension	12.51	55.76	1.52
2	Hypertension	==>	Polyarthrosis	12.51	34.06	1.52
3	Dyslipidemias	==>	Hypertension	12.36	65.31	1.78
4	Hypertension	==>	Dyslipidemias	12.36	33.65	1.78
5	Osteoporosis	==>	Hypertension	9.88	54.53	1.48
6	Hypertension	==>	Osteoporosis	9.88	26.88	1.48
7	Diabetes	==>	Hypertension	9.70	70.27	1.91
8	Hypertension	==>	Diabetes	9.70	26.40	1.91
9	Gastritis	==>	Hypertension	7.49	43.31	1.18
10	Hypertension	==>	Gastritis	7.49	20.38	1.18
11	Cataract	==>	Hypertension	7.46	62.11	1.69
12	Hypertension	==>	Cataract	7.46	20.31	1.69
13	Osteoporosis	==>	Polyarthrosis	6.64	36.68	1.63
14	Polyarthrosis	==>	Osteoporosis	6.64	29.60	1.63
15	Joint disorders	==>	Hypertension	6.00	53.56	1.46
16	Hypertension	==>	Joint disorders	6.00	16.32	1.46
17	Dyslipidemias	==>	Polyarthrosis	6.00	31.67	1.41
18	Polyarthrosis	==>	Dyslipidemias	6.00	26.72	1.41
19	Diabetes	==>	Dyslipidemias	5.55	40.18	2.12
20	Dyslipidemias	==>	Diabetes	5.55	29.30	2.12
