# Correction: Lee et al. Patterns of Multimorbidity in Adults: An Association Rules Analysis Using the Korea Health Panel. *Int. J. Environ. Res. Public Health* 2020, *17*, 2618

**DOI:** 10.3390/ijerph182111278

**Published:** 2021-10-27

**Authors:** Yoonju Lee, Heejin Kim, Hyesun Jeong, Yunhwan Noh

**Affiliations:** 1 College of Nursing, Pusan National University, Yangsan 50612, Korea; lyj@pusan.ac.kr; 2 Department of Nursing, The Graduate School, Pusan National University, Yangsan 50612, Korea; pointsun@naver.com; 3 Department of Statistics, The Graduate School, Pusan National University, Busan 46241, Korea; shepd8516@naver.com

## Figure Correction

The authors have noticed an inadvertent error in our article, ‘‘Patterns of Multimorbidity in Adults: An Association Rules Analysis Using the Korea Health Panel” [1].

The published Figure 2b is a result of a network analysis regarding men, but it should be changed to one regarding women. We have attached a corrected version of Figure 2. This error does not change the scientific conclusion of the article in any way.

The authors would like to apologize for any inconvenience caused.

## Figures and Tables

**Figure 2:**
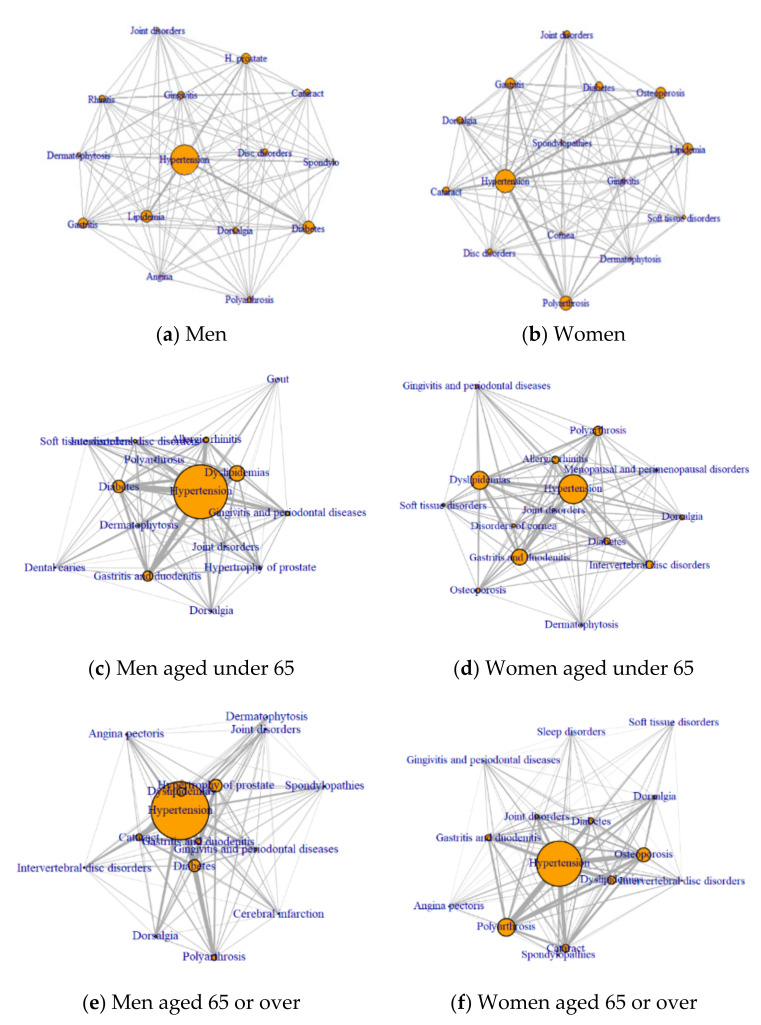
Network of frequent disease based on centrality by gender and age. (**a**) Men; (**b**) women; (**c**) men aged under 65; (**d**) women aged under 65; (**e**) men aged 65 or older; and (**f**) women aged 65 or older.
